# Mutations in Polymerase Genes Enhanced the Virulence of 2009 Pandemic H1N1 Influenza Virus in Mice

**DOI:** 10.1371/journal.pone.0033383

**Published:** 2012-03-15

**Authors:** Wenfei Zhu, Yun Zhu, Kun Qin, Zaijiang Yu, Rongbao Gao, Huiyan Yu, Jianfang Zhou, Yuelong Shu

**Affiliations:** Chinese National Influenza Center, National Institute for Viral Disease Control and Prevention, China CDC, Beijing, People's Republic of China; University of Edinburgh, United Kingdom

## Abstract

Influenza A virus can infect a wide variety of animal species with illness ranging from mild to severe, and is a continual cause for concern. Genetic mutations that occur either naturally or during viral adaptation in a poorly susceptible host are key mechanisms underlying the evolution and virulence of influenza A virus. Here, the variants containing PA-A36T or PB2-H357N observed in the mouse-adapted descendants of 2009 pandemic H1N1 virus (pH1N1), A/Sichuan/1/2009 (SC), were characterized. Both mutations enhanced polymerase activity in mammalian cells. These effects were confirmed using recombinant SC virus containing polymerase genes with wild type (WT) or mutant PA or PB2. The PA-A36T mutant showed enhanced growth property compared to the WT in both human A549 cells and porcine PK15 cells *in vitro*, without significant effect on viral propagation in murine LA-4 cells and pathogenicity in mice; however, it did enhance the lung virus titer. PB2-H357N variant demonstrated growth ability comparable to the WT in A549 cells, but replicated well in PK15, LA-4 cells and in mice with an enhanced pathogenic phenotype. Despite such mutations are rare in nature, they could be observed in avian H5 and H7 subtype viruses which were currently recognized to pose potential threat to human. Our findings indicated that pH1N1 may adapt well in mammals when acquiring these mutations. Therefore, future molecular epidemiological surveillance should include scrutiny of both markers because of their potential impact on pathogenesis.

## Introduction

Influenza A viruses are highly infectious to a variety of mammalian and avian species. Individual viruses are host-specific; however, interspecies transmission of influenza A viruses is not uncommon. Many evidences suggested that genetic mutations in viral proteins, including envelope hemagglutinin (HA) and neuraminidase (NA) glycoproteins [1], nonstructural proteins NS1 [2], [3] and PB1-F2 [4], and the polymerase complex, occur during viral host adaptation and result in enhanced virulence. Of these identified gene markers, viral RNA-dependent RNA polymerase (RdRp), comprising the PB2, PB1, and PA subunits, performs important functions during flu pathogenesis. An avian-like PB1 or its introduction into human-adapted influenza virus might provide a replicative advantage to the new virus for establishing infection in a new host specie, for example, the 1918, 1957 and 1968 pandemic influenza viruses [1], [5]–[8]. Avian viruses with lysine at position 627 in PB2 replicate efficiently in human cells [9], [10] and exhibit higher pathogenicity in mice [11]–[13]. Another amino acid substitution at position 701 in PB2 was involved in the increased lethality of duck-origin H5N1 and avian H7N7 viruses in a mouse model [14]–[16]. Furthermore, both of these PB2 mutations contribute to the transmission of H5N1 virus in guinea pigs [12], [14], [17]–[19]. A mutation close to the cap-binding region of PB2, T271A, may also enhance viral polymerase activity in mammalian cells [20]. Both PB2 and PB1 were found to be responsible for the high virulence of human H5N1 virus in mice and ferrets [21]. Additionally, recent findings suggested that the PA gene of H5N1 viruses is involved in increased virulence to both avian and mammalian hosts [22], [23].

The 2009 pandemic influenza A (H1N1) virus is a swine-origin reassortant with human, avian and swine influenza virus genes. In particular, the polymerase contains PB2 and PA of avian origin, PB1 derived from human viruses and NP from the classical swine lineage. This novel virus was transmitted efficiently from human to human and was more pathogenic than seasonal influenza viruses in both the naive pediatric population and animal models [24]–[28]. However, point mutations of known molecular markers, such as PB2 E627K / D701N/ E677G, PB1-F2 N66S, and PA T97I [15], [29]–[32], produced no significant change in pathogenicity when incorporated in pH1N1 individually. Dual receptor-binding preference, as well as the interaction of viral polymerase components with cellular factors, was found to account for the enhanced lethality of pH1N1 adapting in mice after serial lung-to-lung passage [33], [34]. The D222G substitution in pH1N1 HA, occurring either naturally or as a mouse-adaptation, was associated with severe or fatal disease in mice by altering cell tropism [35], [36]. PB2-590/591 (SR) polymorphism were found to help pH1N1 overcome host restriction by enhancing viral replication [15], [37] and PB2-E158G was a determinant mutation in the adaptation of avian PB2 genes of pH1N1 in mammals [30]. Synergistic effect of mutations in PA (F35L) and HA (D222G and K163E) was recently reported in a mouse-adaptive pH1N1 virus [38]. Mutations in PA, HA and NP have been identified to be related to viral adaptation of pH1N1 to a new host [39].

One or multiple mutations in HA (at position 155, 163, 222 or 223), NA (at position 63), and polymerase (at position 357 of PB2 or 36 of PA ) were found in our recent work on the adaptation of pH1N1 virus, A/Sichuan/1/2009(SC) after 15 passage times in mice [40]. The mutants with single polymerase mutation of PA-A36T or PB2-H357N were found in mouse-adapted descendants of SC. Of 5,875 PA and 5,633 PB2 sequences analyzed up to Jan 30^th^ 2012, less than 1% (including H5 and H7 subtypes) carried these mutations in nature. However, little is known about the impacts of these two rare mutations on virus properties. In this study we fully characterized the variants containing substitution PA-A36T or PB2-H357N in *vitro* and in *vivo*.

## Materials and Methods

### Cell culture

Human embryonic kidney (293T) cells, human type II alveolar epithelial (A549) cell, Madin-Darby canine kidney (MDCK), porcine kidney (PK15) and mouse lung adenoma (LA-4) cells were obtained from the American Type Culture Collection. 293T and A549 cells were maintained in Dulbecco's modified Eagle's medium (DMEM; Invitrogen, Carlsbad, CA, USA) and MDCK and PK15 were in minimum essential medium (MEM; Invitrogen, Carlsbad, CA, USA), respectively, supplemented with 10% fetal bovine serum (FBS; Invitrogen), glutamine (2 mM; Invitrogen), HEPES (10 mM; Invitrogen), penicillin (100 units/ml), and streptomycin (100 µg/ml; Invitrogen). LA-4 cells were propagated in Ham's F12K medium (Sigma, Saint Louis, MO, USA) with 2 mM L-glutamine and 15% FBS.

### Site-Directed Mutagenesis

All eight gene segments of influenza virus A/Sichuan/1/2009 (H1N1, SC_WT) were amplified by RT-PCR and cloned into a modified version of the bidirectional expression plasmid pCQI, derived from pHW2K. Mutations(PA-A36T and PB2-H357N) were introduced into the plasmids to generate mutant segments using a QuikChange™ Site-Directed Mutagenesis Kit (Stratagene, La Jolla, CA, USA), following the manufacturer's instructions. The presence of the introduced mutation and the absence of additional unwanted mutations were verified by sequencing of the whole cDNA.

### Generation of recombinant viruses and virus titration

Recombinant viruses SC_WT and SC_PA-A36T/ SC_PB2-H357N were generated by reverse-genetics method as described as previously [41]. In general, eight plasmids containing the dsDNA representing each gene segment were co-transfected into a 293T/MDCK co-culture monolayer. The identity of propagated mutant viruses was ascertained by sequencing amplicons of each viral gene segment by RT-PCR. Viral titrations were determined using MDCK cells, and the tissue culture infectious dose affecting 50% of the cells (TCID_50_) was calculated using the Reed–Muench formula [42].

### Mini-genome replication assay

polI-Gluc (0.1 µg) [43] were co-transfected with expression plasmids encoding PB2, PB1, PA, and NP into 293T, A549 or PK15 cells (3×10^4^ /well in 96-well plates) using the PolyFect (Qiagen, Valencia, CA, USA) reagent, according to the manufacturer's instructions. Mock transfections were performed with polI-Gluc alone. Gluc activity in supernatants was analyzed using a Gluc assay Kit (New England Biolabs, Beverly, MA, USA). Each sample was determined in triplicates.

### Growth Curves

A549, PK15 and LA-4 cells were infected with rescued viruses at a multiplicity of infection (MOI) of 0.0001, and incubated in the appropriate medium containing 2 mg/L N-p-tosyl-L-phenylalaninechloromethyl ketone-treated (TPCK) trypsin (Sigma, Saint Louis, MO, USA) at 35 or 39°C. At 12, 24, 48, 60, 72, and 96 hour post-inoculation (hpi), supernatants were harvested and virus titer was determined using MDCK cells as described [42].

### Mouse studies

Our mice experiment protocol was approved by the Ethics Committee of National Institute for Viral Disease Control and Prevention, China CDC (IORG0000724). To determine the 50% mouse lethal dose (MLD_50_), groups of five 6-week-old female BALB/c mice obtained from Vital Reviver, PRC, were anesthetized with isoflurane and intranasally inoculated with 50 µl of 10-fold serial dilutions of viruses in phosphate-buffered saline (PBS). The MLD_50_ values were calculated by the method of Reed-Muench [42] after a 14-day observation period and expressed as TCID_50_ causing half fatality. To determine morbidity and mortality, we inoculated groups of 6-week-old female BALB/c mice intranasally with the wildtype and recombinant viruses at the indicated dose in 50 µl volumes, or mock inoculated with PBS. Body weight was monitored continuously for 14 days. Infected mice with more than 30% body loss were recorded as death cases. To determine virus replication in the lungs of infected mice, mice were inoculated intranasally with 10^4^ TCID_50_ of the indicated viruses and three per group were euthanized at 12, 24, 48, and 72 hpi. The right lobe of the lung was homogenized using an ultra-turrax disperser (IKA, Konigswinter, Germany). Homogenates were collected in 1 ml virus collection medium supplemented with 0.2% BSA fraction V and 1% antibiotic-antimycotic. Virus titers were determined by TCID_50_ assay.

### Statistical analysis

All determinations were performed in triplicate and repeated three times. Data are expressed as means ± SEM. Statistical significance was determined using non-parametric tests and the GraphPad Prism 5 software package (GraphPad Software). A *P* value<0.05 was deemed to indicate statistical significance.

## Results

### SNP analysis at 36 of PA and 357 of PB2

To determine whether the amino acid substitutions observed in SC_PA-A36T and SC_PB2-H357N also exist in field strains, we checked all public available sequences from GenBank. Up to Jan 30^th^ 2012, 5,875 and 5,633 sequences were filtered from initial 14,594 and 14,740 PA and PB2 sequences, respectively, and used for polymorphic analysis. Comprehensive analysis showed that residues at these two positions were highly conserved and 99.5% and 98.8% of PA and PB2 gene contained the same residue as SC_WT, respectively. Amino acid at position 357 in PB2 or 36 in PA displayed polymorphic, and the substitutions similar to SC_PB2-H357N and SC_PA-A36T were rare (Table 1). Of note, these mutations were detected in avian H5 and H7 subtype viruses which were currently recognized to pose potential threat to human (Table 1, Table S1).

**Table 1:** Database search for SC_PB2-H357N or PA-A36T mutation in virus isolates from nature.

Viral gene	Amino acid position	Subtype	Total no. sequences	No. same as SC_WT	No. same as SC_M	No. different
PB2	357	H1	779	776(H)	2(N)	1(Y)
		H3	738	734(H)	3(N)	1(Q)
		H5	695	679(H)	—	16(Y)
		H9	286	279(H)	—	7(Y)
		Others*	3135	3133(H)	2(N; H4N2, H10N7)	—
PA	36	H5	1816	1804(A)	12(T)	1(V)
		H7	123	122(A)	1(T)	—
		H1	185	180(A)	—	5(S)
		Others*	3751	3947(A)	4(T; H4N6, H6N9, H9N2, H11N8)	—

Polymorphisms of PA-36 and PB2-357 in influenza virus isolates were assessed and compared with our findings. The data shown are the number of sequences of different influenza subtypes in the NCBI database, the number of sequences of identical same amino acid composition as SC_WT and SC_M, and the number of sequences with a different amino acid at the same position (with the amino acids shown in parentheses). SC_M, SC_PB2-H357N/PA-A36T; Others*, subtypes which were not listed above; —, not applicable.

### RNP complex with PA-A36T or PB2-H357N mutation enhanced the polymerase activity at different temperatures

**Figure 1:**
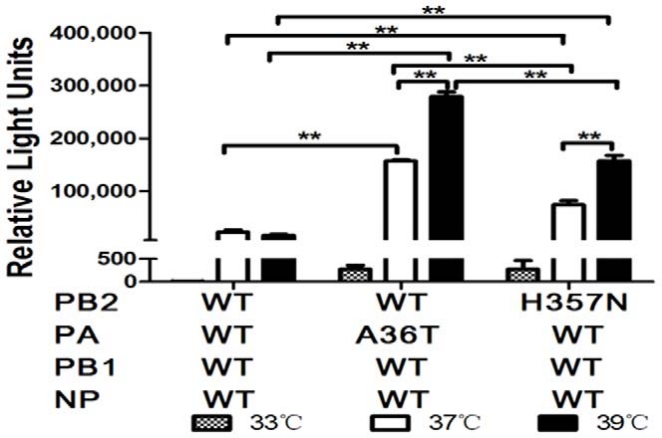
Viral RNA polymerase activity of SC_WT, SC_PA-A36T and SC_PB2-H357N in 293T cells cultured at different temperatures. Luciferase-based minigenome reporter assays were used to measure polymerase activity in 293T cells at 33, 37, or 39°C. Cells were co-transfected with Gluc reporter plasmid and expression plasmids PB1 and NP, PA, and PB2 (WT or PA-A36T, PB2-H357N mutants) to generate different viral RNPs. After culturing at 33, 37, or 39°C for 24 h, *Gaussia* luciferase production was measured. Results are presented as mean ± SEM and are representative of three determinations. **, *p*<0.001, as determined by *t*-test.

In order to investigate whether the PA-A36T or PB2-H357N mutations affect the enzymatic activity of RdRp, we analyzed the reconstituted ribonucleoprotein complexes polymerase activities (composed of PB2, PB1, PA, and NP) in 293T cells at different temperatures using a mini-genome replication assay as described previously [43]. The temperatures 33, 37, and 39°C were used to approximate the conditions under which the influenza virus polymerase would work in different hosts as previously described [44]. As shown in Fig. 1, the polymerase activity of pH1N1 variants and WT displayed a temperature-dependent pattern and was highly active at higher temperature, 37 or 39°C but much lower at 33°C. A similar but less marked trend was also reported by Bussey et al [45]. Moreover, remarkably higher activity was observed in both PA-36T and PB2-357N than that of the WT at both 37 and 39°C (*p*<0.001; *n* = 3). Notably, the strongest RNP activity of PA-A36T than PB2-357N and WT was detected at both 37 and 39°C, and was nearly 10-, and 20-fold increase as compared with WT, respectively (*p*<0.001; *n* = 3).

### PA-A36T or PB2-H357N mutant replicated efficiently in human, porcine and murine cells

**Figure 2:**
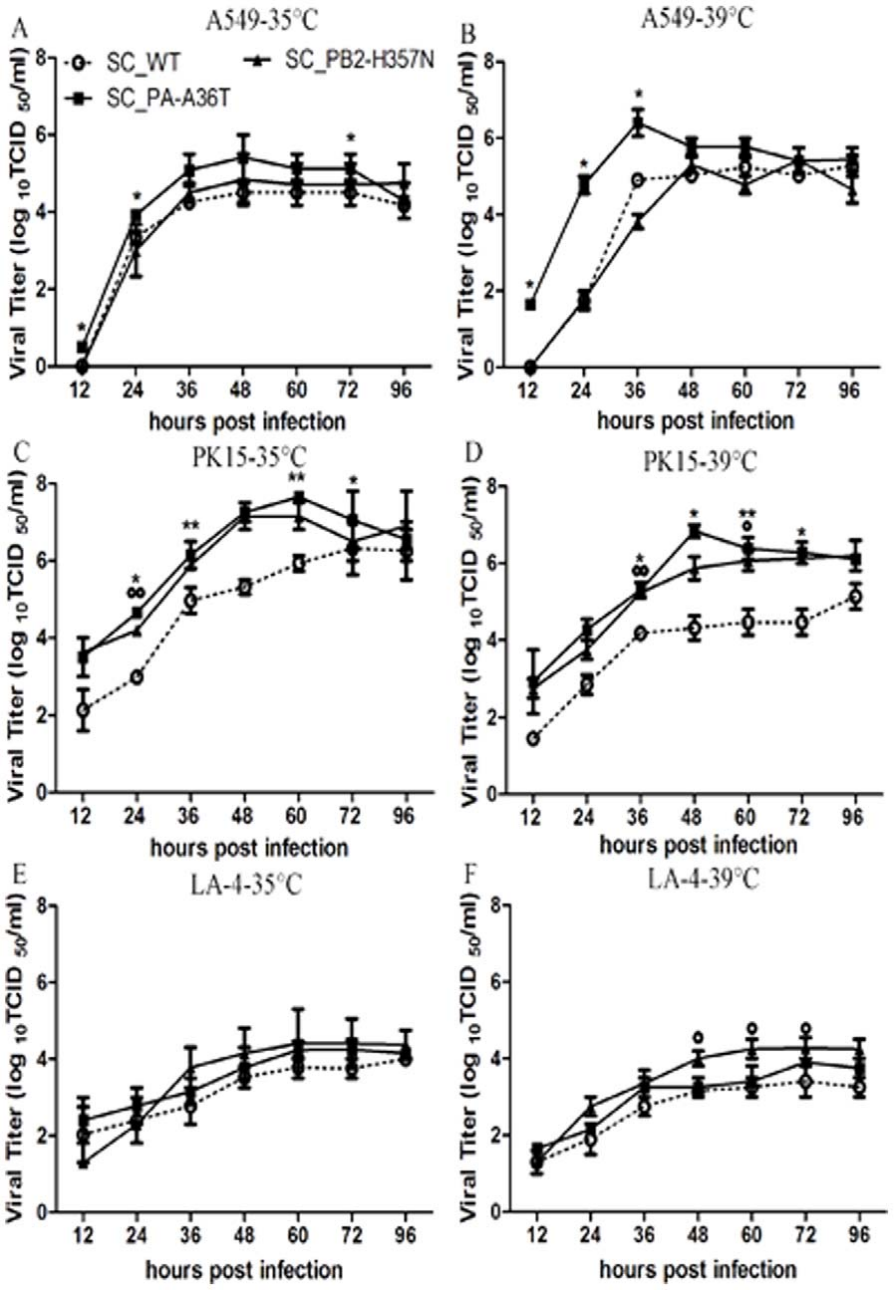
Growth properties of recombinant viruses in human (A, B), porcine (C, D) and murine cells (E, F). Confluent monolayer of A549, PK15 and LA-4 cell lines were inoculated with SC_WT, SC_PA-A36T or SC_PB2-H357N virus at MOI of 0.0001. Culture supernatants were harvested at 12, 24, 48, 60, 72 and 96 hpi at 35(**A, C, E**) or 39°C (**B, D, F**), respectively. Virus titers were determined by TCID_50_ assay using MDCK cells. Results are presented as mean ± SEM and are representative of three determinations. *, °, *p*<0.05, when comparing SC_PA-A36T and SC_PB2-H357N with SC_WT respectively, as determined by a *t*-test of TCID_50_ values. **, °°, *p*<0.001, as determined by *t*-test.

Our ongoing study was to address the issues of the contribution of adaptive mutations to viral replication and the effects of temperature on the mutants during viral infection. Multi-cycle growth assay of recombinant viruses containing PA-A36T or PB2-H357N mutation was performed in human, porcine and murine epithelial cell lines at 35 or 39°C at a MOI of 0.0001. As shown in Figure 2, all the viruses replicated efficiently in the tested cells but were relatively attenuated in murine cells as previously reported elsewhere [38]. The virus titer progressively increased and peaked around 10^5^–10^7^ TCID_50_/ml at 48 hpi. in the human lung epithelia cell A549 and porcine kidney cell PK15. The mutation of PA-A36T exhibited significantly elevated growth ability in both A549 and PK15 cells with virus titers of more than 10-fold higher than those of SC_WT at 24 hpi and the trend maintained throughout the time course (Fig. 2A, B, C, D, *p*<0.001; *n* = 3). Significantly higher virus titer of SC_PB2-H357N mutant than SC_WT was found in PK15 cells at 24 hpi. and the titer maintained at a higher level at following time points although the differences did not reach statistic significance (Fig. 2C, D). In LA-4 cells, statistical differences between them were found from 48 to 60 hpi at 39°C (Fig. 2F, *p*<0.05; *n* = 3). In consistent with the polymerase activity profile at different temperatures (Fig. 1), the mutants displayed advantageous growth capability at a higher temperature (Fig. 2).

### Recombinant viruses containing PA-A36T or PB2-357N mutation enhanced virulence in mice

**Figure 3:**
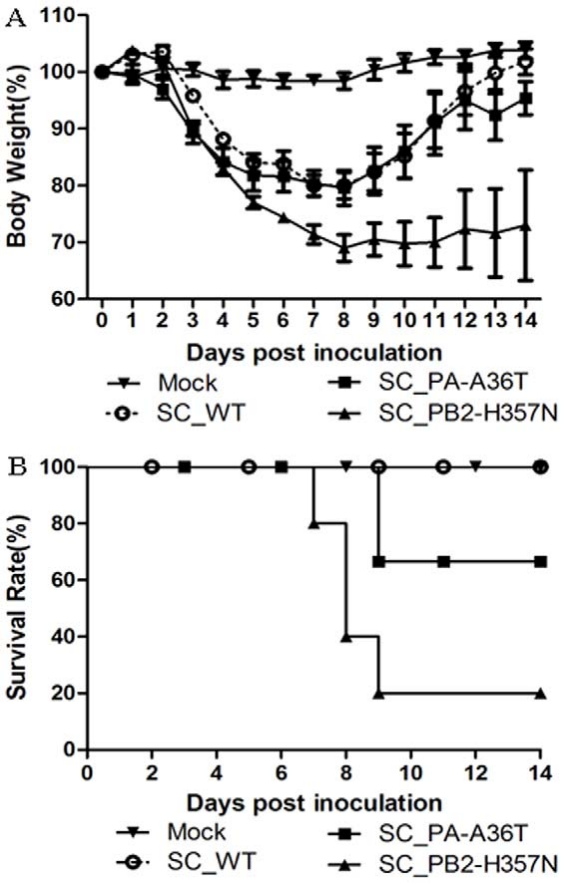
Pathogenicity of SC_WT, SC_PA-A36T, and SC_PB2-H357N viruses in mice. Six-week-old female BALB/c mice (*n* = 5/group) were inoculated intranasally with 50 µl containing 10^4^ TCID_50_ of the recombinant viruses SC_WT, SC_PA-A36T, and SC_PB2-H357N, or PBS (mock). (**A**) Morbidity was assessed by weight changes over a 14-day period and is graphed as a percentage of the average weights on the day of inoculation (day 0). The average body weight change of each group is shown with error bars representing SEM (+/−). (**B**) Mortality associated with infection with the recombinant viruses SC_WT, SC_PA-A36T, and SC_PB2-H357N was also examined.

To elucidate the contribution of the PB2-H357N and PA-A36T mutations to virulence in mice, we used recombinant viruses to determine the MLD_50_. In contrast to SC_PA-A36T and SC_WT (10^5^ and 10^5.5^ TCID_50_ causing 50% mice death, respectively), PB2-H357N has an MLD_50_ of 10^3.5^ TCID_50_. Mice were inoculated intranasally with 10^4^ TCID_50_ of SC_WT, SC_PB2-H357N, and SC_PA-A36T. Approximately 30% weight loss was detected in SC_PB2-H357N-infected mice together with an 80% mortality rate (Fig. 3A & B). In contrast, the SC_PA-A36T and SC_WT viruses caused around 20% weight loss with no significant difference (Fig. 3A). However, the former had a mortality rate of 20%, while all mice inoculated with SC_WT survived (Fig. 3B).

**Figure 4:**
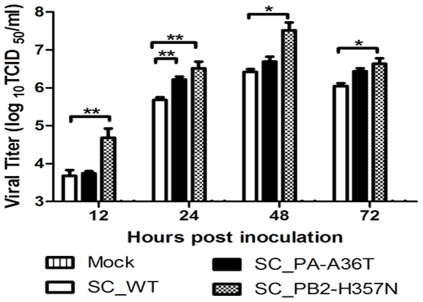
Analysis of viral replication efficiency in the respiratory tracts of mice. Six-week-old female BALB/c mice (*n* = 3/group/time-point) were inoculated intranasally with 50 µl containing 10^4^ TCID_50_ of SC_WT, SC_PA-A36T, and SC_PB2-H357N. Animals were euthanized at 12, 24, 48 and 72 hpi. The right lung of each animal was homogenized in PBS (1 ml) and then centrifuged. Viral titers in the supernatant from lung homogenates were determined by TCID_50_ assay. Results are presented as mean ± SEM and are representative of three determinations. *, *p*<0.05 and **, *p*<0.001, as determined by *t*-test.

To determine if SC_PB2-H357N/SC_PA-A36T replicated more efficiently than SC_WT virus *in vivo*, we inoculated mice with 10^4^ TCID_50_ of each virus and analyzed virus titers in lung at various time points post inoculation (Fig. 4). In line with viral replication in LA-4 cells, titer of SC_PB2-H357N was significant higher than that of WT at all the tested time points, and peaked (>10^7^ TCID_50_/ml) at 48 hpi (Fig. 4). Similar to viral replication in A549 and PK15 cells , significantly higher titers of SC_PA-A36T were detected at early stage, i.e., 24 hpi. (*p*<0.001; *n* = 3), while did not differ from that of SC_WT at any following time points. Our study showed that the SC_PB2-H357N mutant displayed marked replication activity in mice and caused severe infection and high mortality.

## Discussion

The polymerase complex of influenza A virus, known to function in many aspects of viral replication and to interact with host factors, therefore, play a critical role during viral adaptation process. Unlike 1957 H2N2 and 1968 H3N2 pandemic viruses caused by reassortants between avian and human influenza viruses, 1918 H1N1 influenza virus derived its polymerase genes directly from avian source before the pandemic emergence. The finding that ten consensus residues observed in 1918 H1N1 polymerases and those from other mammalian viruses is indicative of its human adaptation [46]. Several mutations in polymerase genes associated with adaption of H5N1 virus in mammalian hosts have been identified from human infection cases and mice model, however, their introduction into pH1N1 did not exhibit altered virulent phenotype in mice [15], [29]–[32]. Therefore, the mutation pattern of the polymerase complex required for viral adaptation and virulence to a new host as well as underlying mechanisms remains to be investigated. To decode the factors contributing to adaptation and virulence of pH1N1 and to assess its potential risk to human health, an experimental evolutionary study was conducted and the polymerase mutations PA-A36T and PB2-H357N, were observed in the mouse-adapted descendants of pH1N1 virus-A/Sichuan/1/2009 (SC).

The variants with the detected mutations were characterized in this study. Both mutations enhanced the polymerase activity remarkably. Furthermore, the activity of WT and mutations displayed a temperature-dependent pattern and highly active at higher temperature. The mutations, particularly PA A36T, exhibited the strongest up-regulation effect at 39°C than that of either 33°C or 37°C. Interestingly, the viruses containing the mutations demonstrated similar temperature-dependent effects on viral replication in mammalian cells from different species. Although the mutants showed varied replication properties in different cells, better growth of the mutants with PA-36T or PB2-357N than WT in mammalian cells as well as increased RNP activity are indicative of their mammalian adaptation. A synergistic effect on polymerase activity rendered by dual PA-36T and PB2-357N substitutions was observed (data not shown) and the mutant containing PA-A36T and PB2-H357N accompanied with HA-D222G was detected in our mice-adaptive experiments. However, the variant with dual mutations, PA-36T and PB2-357N, showed nearly identical virulence to that of WT in mice model (data not shown). Of note, it was recently identified that synergistic actions of HA D222G, K163E and PA F35L in pH1N1 result in an enhanced pathogenicity in mice model [38]. Therefore, the virulence of influenza virus may depend on numerous factors including cell tropism, viral infectivity or replicate activity and so on. Our data together with other previous reports, clearly showed that a high level of polymerase activity is not the sole reason for increased viral replication or virulence [14], [15], [30], [32], [47], [48]. It is likely that a subtle functional optimum of viral proteins during virus infection is required for high virulence.

As a part of hetrotrimeric RdRp, PA functions in diverse aspects of the complex including endonuclease, protease and viral RNA binding [49]–[52]. The position 36 in the N-terminal domain of PA, which contains the endonuclease activities and was required for cap snatching [53]. Thus, substitution of A36T in PA may affect viral transcription and replication. Previous studies have showed that the PA subunit is a virulence factor for H5N1 and pH1N1 influenza A virus [15], [45], [54], [55]. Although the A36T in PA in our study did not remarkably alter viral pathogenicity in mice, it contributed significantly to RNP activity and viral replication in mammalian cells, especially in PK15 cells. Since pig is the hypothesized ‘mixing vessel’ for generating novel reassortants or pandemic flu viruses, the superior growth properties of SC_PA-A36T in PK15 cells implies a potential threat of the variants carrying this mutation and required its monitoring in future work.

Residues 242–252(N-site), 318–483(C-site) and 533–577 are thought to be the cap-binding domain on PB2. Position 357 is located at the C-terminal of strand β4 which was mapped to positions 318 to 483 and was involved in viral transcription via snatching the cap of host mRNA. H357A mutation could decrease PB2's cap binding activity and thus reduce mRNA transcription while H357W showed an enhanced binding to m^7^GTP [56]. Theoretically, asparagines (N) at position 357 of PB2 observed here might attenuate the binding to m7GTP as compared with W or Y. Nonetheless, enhanced polymerase activity and higher virulence in mice were detected.

Systematic analyzing PA and PB2 sequences from all available data of GenBank showed high conservation of the both residues in field isolates and less than 1% viruses possessed mice adaptive mutations. Such mutations exclusively presented in avian viruses including H5 and H7 subtypes. Although whether the mutation pattern for pH1N1, observed here, represents a preferred viral strategy to gain virulence is still unclear, careful monitoring these virulence enhancing mutations is critical important for pandemic preparedness and response due to increasing interspecies transmission events of pH1N1 virus into other hosts have been documented [57]–[61]. In summary, our results show that the amino acid 36T in PA and 357N in PB2 contribute to polymerase activity and viral replication of pH1N1 in mammalian cells. The amino acid 357N in PB2 also plays a role in viral pathogencity in mice model.

## Supporting Information

Table S1 **Natural isolates with PB2-357N or PA-36T.**
(DOCX)
