# Supplementary material for: Mutations in Polymerase Genes Enhanced the Virulence of 2009 Pandemic H1N1 Influenza Virus in Mice
Source: PLoS One. 2012 Mar 15;7(3):e33383. doi: 10.1371/journal.pone.0033383 (PMC3305307; doi:10.1371/journal.pone.0033383)
Supplement: Table S1 — Natural isolates with PB2-357N or PA-36T. (DOCX) [file pone.0033383.s001.docx]

**Supplementary data**

**Table S1 Natural isolates with PB2-357N or PA-36T**

| Locations | Field Strains | Subtype |
| --- | --- | --- |
| PB2-357N | A/mallard/Maryland/322/2002 | H1N1 |
|  | A/mallard/Maryland/334/2002 | H1N1 |
|  | A/mallard/Interior Alaska/6MP0272/2006 | H3N8 |
|  | A/mallard/Interior Alaska/6MP0972R1/2006 | H3N8 |
|  | A/green-winged teal/Interior Alaska/6MP0736/2006 | H3N8 |
|  | A/turkey/Minnesota/833/1980 | H4N2 |
|  | A/ruddy turnstone/NJ/238/2004 | H10N7 |
| PA-36T | \| A/green-winged teal/Ohio/1324/2005 \| \| --- \| | H4N6 |
|  | A/chicken/Hubei/wj/1997 | H5N1 |
|  | A/chicken/Henan/210/2004 | H5N1 |
|  | A/chicken/Nigeria/SO494/2006 | H5N1 |
|  | A/tern/South Africa/1959 | H5N1 |
|  | A/wild duck/Guangdong/314/2004 | H5N1 |
|  | A/tree sparrow/Henan/1/2004 | H5N1 |
|  | A/chicken/Queretero/7653-20/1995 | H5N2 |
|  | A/chicken/Queretero/22019-853/1996 | H5N2 |
|  | A/chicken/Queretaro/14588-19/1995 | H5N2 |
|  | A/chicken/Henan/1362/2006 | H5N2 |
|  | A/tern/South Africa/1961 | H5N3 |
|  | A/avian/New York/Sg-00418 | H5N8 |
|  | A/mallard/New Zealand/1365-350/2005 | H6N9 |
|  | A/environment/New York/23165-4/2005 | H7N2 |
|  | A/chicken/Heilongjiang/35/00 | H9N2 |
|  | A/environment/Delaware/234/2005 | H11N8 |
